# A snapshot of nutritional recommendations and management practices adopted by feedlot cattle nutritionists in Brazil in 2023

**DOI:** 10.3389/fvets.2025.1518571

**Published:** 2025-06-04

**Authors:** Julia Gabrielle Monsalve, Danilo Domingues Millen

**Affiliations:** School of Agricultural and Veterinary Sciences, São Paulo State University (UNESP), São Paulo, Brazil

**Keywords:** beef, cattle, feedlot, nutrition, practices, survey

## Abstract

**Introduction:**

This survey was conducted to provide an overview of the nutritional strategies and management practices used by feedlot nutritionists in Brazil.

**Methods:**

The online questionnaire consisted of 100 questions and covered a wide range of topics, including information about nutritionists, ingredients used in finishing diets, feeding strategies, management practices, challenges in applying nutritional recommendations, and animal performance results.

**Results:**

The 36 participating nutritionists were responsible for 6,082,698.52 animals, representing 79.8% of the cattle from feedlots slaughtered in 2023. Corn remains the preferred grain choice among nutritionists, with 91.7% of the responses. Regarding the grain processing used, the high-moisture re-hydrating and storage was chosen by 34.3% of participants, overcoming other methods of grain processing, such as grinding, which reflects the continuous search for starch optimization in the Brazilian feedlots. Coproducts have been widely used in finishing diets in Brazilian feedlots, with 92.7% of nutritionists’ clients reporting its use, highlighting a focus on sustainable and economically viable practices. In this context, dried distillers’ grains have established a strong position in the Brazilian market as an important coproduct source to meet protein demands, being chosen as the primary protein source for the first time in feedlot history in Brazil. This study also emphasizes the widespread use of technologies at the operational level, with 80.8% of feedlots reporting the use of truck-mounted mixers and 81.4% adopting feed deliveries by pen. These practices have enabled feedlot nutritionists to increase the energy level of finishing diets, resulting in higher use of peNDF (85.3%) and more accurate monitoring of the amount of fiber available for rumination. Additionally, animal welfare practices have been implemented, such as shading in pens (18.8%) and the use of sprinklers (53.1%).

**Conclusion:**

The data collected point to a notable change in the diets and management practices of Brazilian feedlots. This evolution reflects an adaptation to the needs of the sector, as well as a growing commitment to efficiency and sustainability. These trends point to a promising future for feedlots in Brazil and highlight the continued need for research and innovation to drive feedlot operations to advanced practices.

## 1 Introduction

The Brazilian livestock industry, a major player in global beef markets, reached a milestone in 2023 with a record 202 million cattle heads, reflecting a 3.3% increase in the herd (22). Despite the dominance of extensive beef production systems, there’s a noticeable rise in the slaughter of cattle from feedlots, constituting 18.2% of the 42.3 million total slaughters in 2022 (22). This shift towards more intensive practices highlights the evolving nature of the Brazilian feedlot industry. In this scenario, research projects have been crucial in understanding critical points to control and advance the recent Brazilian feedlot industry. Inspired by USA surveys, the first survey with Brazilian feedlot cattle nutritionists took place in 2009 (1), marking 15 years of data collection to monitor the feedlot industry development in Brazil.

The four surveys conducted prior to this one, in 2009 (1), 2013 (2), 2016 (3), and 2019 (4) revealed significant improvements in feeding management practices, resulting in higher energy content of finishing diets in Brazilian feedlots. These practices include the widespread adoption of technologies like truck-mounted mixers, enabling scheduled feed deliveries for most feedlots in Brazil. This technological advancement enhances operational efficiency and feeds resource control. Previous surveys indicated increased use of physically effective NDF and a boost in the energy content of Brazilian feedlot diets through higher grain and fat inclusions. This has contributed to animals achieving heavier slaughter weights. These improvements underscore the proactive role of the feedlot nutrition industry in using the survey findings to plan and implement enhancements in the production system. Researchers have formulated hypotheses and conducted nutritional studies to improve feed efficiency and, consequently, the profitability of Brazilian feedlots. The dynamic nature of the industry, marked by continuous improvement and adaptation, positions Brazil as a key player in the evolving landscape of global beef production.

However, based on the four surveys conducted previously, some critical issues still required the attention of feedlot nutritionists over the last 4 years. These include optimizing starch use by adopting better methods for grain processing, and the high percentage of feedlots using continuous feed delivery (about 30%), along with bunk management that results in more than 3% orts.

Therefore, this survey was designed to (1) provide a snapshot of the current nutritional recommendations and management practices adopted by Brazilian feedlot cattle nutritionists and compare it with the previous four surveys conducted in Brazil, and (2) provide insights to control critical issues and collaborate to research development to increase efficiency, in general, and productivity of feedlot operations in Brazil.

## 2 Materials and methods

The study was conducted at São Paulo State University (UNESP), Jaboticabal campus, Brazil. Since no animals were used in this study, approval from the Ethics Committee for Animal Use (CEUA) at UNESP, Jaboticabal campus, was not required. Additionally, following Article 1, sole paragraph of Resolution 510/16 of the National Health Council of the Ministry of Health in Brazil: “There is no need to submit a project to the Ethics Committee when the interviewed participants are not identified, and the research has public access.”

### 2.1 Nutritionists and data collection

Data collection for this study followed methods like those employed by Vasconcelos and Galyean (5) and Silvestre and Millen (4), who used SurveyMonkey^1^ as the research instrument and online survey platform. Forty-nine feedlot cattle nutritionists were invited to participate in this survey. These nutritionists were selected based on their current role in the feedlot industry and for representing typical cattle feeding practices in different states of Brazil. Nutritionists were first contacted by email about their interest in participating. A second email, containing a unique identification number and instructions to respond to the questionnaire, was sent to 44 nutritionists who agreed to participate. Nutritionists were guaranteed to remain anonymous, and then their identity was not disclosed in this study. Thirty-six nutritionists ultimately completed the survey.

The questionnaire was hosted on the mentioned website and remained accessible to participating nutritionists for two months. During this time, participants were encouraged to respond through reminders sent to their registered email addresses. To ensure the quality and integrity of the data, periodic contacts were made via email and phone, providing support and clarifying any questions related to questionnaire completion.

### 2.2 Survey questions

The questionnaire developed regarding the nutritional and management practices of Brazilian feedlots consisted of 100 questions, including both open-ended and multiple-choice questions. These questions were categorized into various sections, such as general information (*n* = 11), general information on grains and concentrate recommended in finishing diets (*n* = 13), use of coproducts in finishing diets (*n* = 5), sources and levels of forage (*n* = 5), adaptation methods (*n* = 7), mixers (*n* = 6), feeding management (*n* = 7), animal management and information (*n* = 15), information about diet formulation (*n* = 17), information about sources for nutritional recommendations (*n* = 2), and additional questions and parameters related to animal well-being (*n* = 12). For questions that were not answered by the 36 interviewed nutritionists, the total number (n) of responses was indicated either in the table or in the results section. The terms “primary” and “secondary” were used to describe the most often and second most often used feedstuffs, feed additives, and grain processing methods. The terms DDG and WDG were cited by the nutritionists as sources of protein, fat and coproducts. However, it’s important to highlight that in Brazil, the most common products are DDGS (dry bran/fiber plus soluble) and WDGS (wet bran/fiber plus soluble). We advise readers to take this information into consideration when reading this article.

### 2.3 Data analysis

The data from this survey was analyzed following the methods of Vasconcelos and Galyean (5) and Silvestre and Millen (4). The data collected were tabulated in an Excel spreadsheet (Microsoft, Redmond, WA, USA), where the number of responses, mode, average values, maximum values, and minimum values were calculated when appropriate.

## 3 Results

### 3.1 General information

In 2023, the 36 participating nutritionists were collectively responsible for 6,082,698 animals. Consulting companies employed 25.0% of the professionals surveyed, while 33.33% were affiliated with cattle nutrition companies. Additionally, 22.2% were independent consultants, 11.1% held positions as feedlot managers, 2.8% were associated with a university, and 2.8% were researchers. Regarding professional experience, most nutritionists interviewed (91.7%) have more than 10 years of practice, while 5.6% have between 5 and 8 years, and 2.8% have 2 to 5 years of experience. Of the respondents, 5.6% held a Bachelor of Science degree, 38.9% had a specialization course in cattle nutrition, 30.6% held a Master of Science degree, 22.2% had a Ph.D. degree, and 2.8% had post-doctoral qualifications. Most participating nutritionists (49.0%) indicated that their educational background was obtained in the state of São Paulo, followed by Minas Gerais (17.7%), Goiás (7.8%), Paraná (7.8%), Mato Grosso (5.9%), Tocantins (2.0%), and other states (9.8%). This survey findings indicate diversity in the average size of feedlots attended, with 17.1% of nutritionists serving those with less than 1,000 heads, 28.6% servicing feedlot operations ranging from 1,001 to 5,000 heads, 22.9% assisting feedlots containing between 5,001 to 10,000 heads, 11.4% reported to be consulting to feedlots operations between 10,001 to 20,000 heads, and 20.0% of the nutritionists declared to consult for feedlots with more than 20,000 heads. In addition, the average interval between each client’s visits was 35.4 days on average. Nutritionists’ clients were spread across different Brazilian states, including São Paulo (*n* = 26), Goiás (*n* = 18), Mato Grosso do Sul (*n* = 21), and Bahia (*n* = 9), among others. Some nutritionists have clients in countries such as Paraguay (*n* = 10), Argentina (*n* = 3), Uruguay (*n* = 3), United States (*n* = 2), Bolivia (*n* = 3), Mexico (*n* = 1), Nicaragua (*n* = 1), Russia (*n* = 1), and China (*n* = 1), with an average of 11.8% of their work being in foreign countries.

### 3.2 General information on grains and concentrate recommended for finishing diets by Brazilian nutritionists

#### 3.2.1 Grains

Corn was the primary grain source cited by 33 nutritionists (91.7%), while sorghum was mentioned by 3 respondents (8.3%). Concerning corn variety, the Flint type was the choice of 33 nutritionists (97.1%), and the Dent type was chosen by only one of the nutritionists interviewed (2.9%). Information on grains is shown in Table 1.

**Table 1:** General information on grains and concentrate recommended for finishing diets by the Brazilian nutritionists.

Item	*N*° of responses^1^	% of responses
Primary grain source used		
Corn	33	91.67
Sorghum	3	8.33
Secondary grain source used	*n* = 35	
Sorghum	30	85.71
Corn	3	8.57
Barley	1	2.86
Wheat	1	2.86
Type of corn used	*n* = 34	
Flint	33	97.06
Dent	1	2.94
Primary grain-processing method		
Finely ground	11	30.56
Coarsely ground	11	30.56
High-moisture harvesting and storage (re-hydrated)	11	30.56
High-moisture harvesting and storage (harvested wet)	2	5.56
Only cracked	1	2.78
Secondary grain-processing method	*n* = 35	
High-moisture harvesting and storage (re-hydrated)	12	34.29
Finely ground	10	28.57
High-moisture harvesting and storage (harvested wet)	8	22.86
Whole-shelled corn grain	2	5.71
Only cracked	1	2.86
Steam-flaking	1	2.86
Coarsely ground	1	2.86
Inclusion level of grains in finishing diet (% of dry matter)		
20–35	3	8.33
36–50	11	30.56
51–65	16	44.44
66–80	5	13.89
81 or more	1	2.78
Inclusion level of concentrate in finishing diet (% of dry matter)		
Less than 55	0	0
56–70	2	5.56
71–80	6	16.67
81–90	26	72.22
91 or more	2	5.56

^1^ Number of responses when nutritionists chose only one answer for a question.

#### 3.2.2 Grain processing methods

For the primary grain-processing method, the high-moisture re-hydrating and storage was the choice of 30.6% (*n* = 11) nutritionists, but also 30.6% (*n* = 11) reported preference for finely ground and other 30.6% (*n* = 11) for coarsely ground. High-moisture harvesting and storage was employed by two nutritionists interviewed (*n* = 2), and only one nutritionist (2.8%) declared that the primary grain-processing method was only cracking.

Concerning the secondary grain-processing method, high-moisture re-hydrating, and storage was the preference of 34.3% (*n* = 12) nutritionists, followed by finely ground (28.6%). Information on grain-processing methods is shown in Table 1. It is noteworthy to mention that the particle size used for corn was 3.1 mm (minimum = 1.5 mm; maximum = 6.0 mm; and mode = 4.0 mm; data not shown).

#### 3.2.3 Average inclusion level of grain and concentrate

Regarding the level of grain inclusion in the finishing diets shown in Table 1, 44.4% of participants (*n* = 16) stand out by recommending the inclusion from 51 to 65% (DM basis), followed by 30.6% nutritionists (*n* = 11) that suggested grain inclusion level from 36 to 50%. For the inclusion level of concentrate ingredients in the finishing diets, 72.2% of the nutritionists (*n* = 26) recommended from 81 to 90% (DM basis), whereas 5.6% (*n* = 2) indicated inclusions of 91% or more.

#### 3.2.4 Feed energy values

Considering the type of energy unit preferred by nutritionists for formulating finishing diets, the results revealed a preference for Total Digestible Nutrients (TDN), which was the choice of 50.0% of the participants (*n* = 18), followed by NEg (*n* = 12, 33.3%), ME (*n* = 5, 13.9%), and non-fibrous carbohydrates (*n* = 1, 2.8%; Table 2).

**Table 2:** Information on energy units and values used for diet formulation by nutritionists surveyed.

Item	*N*° of responses^1^	% of responses	Average level	Mode	Minimum	Maximum
Type of energy unit used to formulate finishing diets, dry matter basis						
Total digestible nutrients (%)	18	50.00	76.76	78.00	70.00	85.00
Net energy for gain (Mcal/kg)	12	33.33	1.28	1.25	1.20	1.43
Metabolizable energy (Mcal/kg)	5	13.89	2.93	2.80	2.80	3.20
Non-fibrous carbohydrates (%)	1	2.78	54.00	-	54.00	54.00
Nutritional models and source of information on energy values	*n* = 32					
BCNRM ou NASEM (2016)	10	31.25				
RLM	9	28.13				
CNCPS – Cornell	6	18.75				
NRC (24)	3	9.38				
BR – Corte	2	6.25				
Private-company system	1	3.13				
AFRC	1	3.13				

^1^ Number of responses when nutritionists chose only one answer for a question. RLM – Ração de Lucro Máximo; CNCPS – Cornell Net Carbohydrate and Protein System; NRC – National Research Council; NASEM – National Academies of Sciences, Engineering, and Medicine; AFRC – Agricultural and Food Research Council.

#### 3.2.5 Nutritional models and sources of information on energy values

The main nutritional model and source of information for feed energy values was the NASEM (6), preferred by 31.3% of the nutritionists interviewed (*n* = 11; Table 2). Nine participants (28.1%) reported the use of RLM (23), six (18.8%) the CNCPS- Cornell, three (9.4%) (24), and two (6.3%) the BR-Corte (25). One nutritionist (3.1%) declared to use a private-company system, and another participant (3.1%) used the Agricultural and Food Research Council (AFRC; 3.1%). About the various sources of general information used by nutritionists, it is noteworthy to mention that the Journal of Animal Science was the choice of 70.0% of the participants, followed by the Revista Brasileira de Zootecnia (RBZ), which was mentioned by 13.3% of the nutritionists. Additional sources of general information included podcasts and Instagram (6.7%), articles (3.3%), symposiums and conferences (3.3%), and DBO, a Brazilian magazine (3.3%; data not shown).

### 3.3 Use of coproducts

Thirty-four nutritionists reported that 92.7% (mode = 100%) of their clients use some type of coproduct in their finishing diets (Table 3). The pelleted citrus pulp was indicated by 26.5% of participants (*n* = 9) as the primary coproduct used in finishing diets, followed by whole cottonseed (20.6%; *n* = 7) and dry distillers’ grains (DDG; 20.6%; *n* = 7). Other coproducts that were mentioned included cottonseed cake (17.7%; *n* = 6), soybean hulls (8.8%; *n* = 3), wet distillers’ grains (WDG; 2.9%; *n* = 1), and corn germ meal (2.9%; *n* = 1). The average level of inclusion of citrus pulp, whole cottonseed, and DDG were 26.1, 14.1, and 25.0% of diet DM, respectively (Table 3). The main secondary coproduct used in the finishing diets cited by the 12 nutritionists (34.3%) was the DDG.

**Table 3:** Concentrate coproducts used in finishing diets by the Brazilian nutritionists.

Item	*N*° of responses^1^	% of responses	Average level	Mode	Minimum	Maximum
Primary concentrate coproduct used	*n* = 34					
Citrus pulp, pellets	9	26.47	26.12 (*n* = 8)	30.00	14.00	35.00
Whole cottonseed	7	20.59	14.14 (*n* = 7)	15.00	5.00	20.00
DDG (Dry Distillers Grains)	7	20.59	25.00 (*n* = 7)	25.00	10.00	45.00
Cottonseed cake	6	17.65	15.67 (*n* = 6)	17.00	10.00	20.00
Soybean hulls	3	8.82	18.33 (*n* = 3)	15.00	15.00	25.00
WDG (Wet Distillers Grains)	1	2.94	45.00 (*n* = 1)	–	45.00	45.00
Corn germ meal	1	2.94	27.00 (*n* = 1)	–	27.00	27.00
Secondary concentrate coproduct used	*n* = 35					
DDG (Dry Distillers Grains)	12	34.29	18.41 (*n* = 12)	20.00	12.00	25.00
Whole cottonseed	6	17.14	12.00 (*n* = 6)	10.00	10.00	15.00
Cottonseed cake	6	17.14	15.50 (*n* = 6)	17.00	9.00	19.00
Citrus pulp, pellets	5	14.29	25.60 (*n* = 5)	–	15.00	42.00
Soybean hulls	4	11.43	19.25 (*n* = 4)	–	10.00	40.00
Corn gluten feed (Refinazil®)	2	5.71	20.00 (*n* = 1)	–	20.00	20.00
Percentage of clients using some type of coproduct in finishing diets	*n* = 34	–	92.71	100.00	68.00	100.00

^1^ Number of responses when nutritionists chose only one answer for a question.

### 3.4 Roughage sources and levels and methods of fiber analysis

#### 3.4.1 Roughage sources and levels

The average inclusion level of roughages in finishing diets (*n* = 33), expressed as a percentage of DM, was 15.7%, ranging from 10.0 to 25.0% (Table 4).

**Table 4:** Roughage sources and levels and fiber analysis methods adopted by Brazilian nutritionists.

Information	*N*° of responses	% of responses	Average level	Mode	Minimum	Maximum
Primary roughage source	*n* = 34					
Corn silage	19	55.88				
Grass silage	7	20.59				
Sugarcane bagasse	3	8.82				
Sorghum silage	1	2.94				
Peanut shells	1	2.94				
Sugarcane silage	1	2.94				
Sweetcorn silage	1	2.94				
Sugarcane silage or grass silage	1	2.94				
Secondary roughage source	*n* = 34					
Grass silage	12	35.29				
Sugarcane bagasse	8	23.53				
Corn silage	8	23.53				
Peanut shells	2	5.88				
Fresh-chopped sugarcane	2	5.88				
Cottonseed pod	1	2.94				
Sorghum silage	1	2.94				
Method of fiber analysis	*n* = 34					
peNDF	29	85.29	13.62 (*n* = 29)	15.00	10.00	18.00
NDF	4	11.76	22.00 (*n* = 3)	–	12.00	30.00
None	1	2.94				
Equipment for determining dry matter in the field						
Air Fryer	21	58.33				
Koster	7	19.44				
Microwave	4	11.11				
Air Fryer + Oven	2	5.56				
Oven	2	5.56				
Average inclusion level of roughage in finishing diets (% of DM):	*n* = 33		15.73	15.00	10.00	25.00

DM – dry matter; peNDF – physically effective neutral detergent fiber; NDF – neutral detergent fiber. ^1^ Number of responses when nutritionists chose only one answer for a question.

Notably, corn silage is the most commonly used source, adopted by 55.9% of the participants (*n* = 19). Second, grass silage was mentioned by 20.6% of the nutritionists interviewed (*n* = 7), followed by sugarcane bagasse (8.8%; *n* = 3). Other sources cited by the nutritionists included sorghum silage, peanut shells, sugarcane silage, sweet corn silage, and a combined option of sugarcane silage or grass silage (Table 4). When considering the second most adopted roughage source, grass silage was cited by 35.3% of the nutritionists (*n* = 12), followed by sugarcane bagasse (23.5%; *n* = 8).

#### 3.4.2 Methods of fiber analysis and determination of dry matter in the field

Thirty-four participants (85.3%) indicated that they use peNDF as their fiber analysis method of choice, while 11.8% (*n* = 4) indicated NDF. One respondent (2.9%) indicated that they do not employ any method for fiber analysis. The average peNDF inclusion level was 13.6% (DM basis), with a range of 10.0 to 18.0% and a mode of 15.0% (*n* = 29). For those who use NDF, the recommended NDF content in finishing diets was 22.0% (*n* = 4), with a minimum of 12.0% and a maximum of 30.0% (DM basis).

Concerning the equipment for determination of dry matter in the field, the Air Fryer was the most adopted method, cited by 58.3% of the respondents (*n* = 21), followed by Koster (19.4%; *n* = 7), Microwave (11.1%; *n* = 4), Air Fryer + Oven (5.6%; *n* = 2), and Oven (5.6%; *n* = 2).

### 3.5 Receiving programs and adaptation methods for cattle

#### 3.5.1 Receiving programs

The feedlot bunk containing a mix of roughage and concentrate was indicated by 16 nutritionists (48.5%) as the most used receiving program for feedlot cattle in Brazil, followed by the feedlot bunk containing only roughage (15.2%; *n* = 5). Five nutritionists (15.2%) reported that they do not use any type of receiving program (Table 5). Nutritionists who reported adopting receiving programs informed that 5 days was the average length of the receiving period (mode = 7.0 days; data not shown).

**Table 5:** Receiving programs and adaptation methods for cattle adopted by Brazilian nutritionists.

Item	*N*° of responses^1^	% of responses
Receiving program	*n* = 33	
Feedlot bunk containing roughage and concentrate	16	48.48
None	5	15.15
Feedlot bunk containing only roughage	5	15.15
Feedlot bunk containing the adaptation diet and hay in the pen	3	9.09
Pasture plus bunk containing concentrate	3	9.09
Pasture	1	3.03
Methods used for adapting cattle to finishing diet	*n* = 34	
Multiple step-up diets	20	58.82
Blending of two diets	7	20.59
Only one diet containing less energy than the finishing diet	5	14.71
Finishing diet limited by quantity	2	5.88
None	0	0.00

^1^ Number of responses when nutritionists chose only one answer for a question.

#### 3.5.2 Adaptation methods

Brazilian nutritionists showed a preference for the adaptation method known as the multiple-step-up diet, which was cited by 58.8% of the respondents (*n* = 20; Table 5). According to the nutritionists that use this method, the initial average level of roughage was 38.8% (DM basis) and the duration of the adaptation period was, on average, 19.3 days (Table 6). The blending of two diets was the second adaptation method of choice, indicated by 20.6% of the participants (*n* = 7); followed by one single diet containing less energy than the finishing diet (14.7%; *n* = 5), and restriction protocol, in which the finishing diet is fed throughout the entire feeding period and limited by quantity during adaptation (5.9%; *n* = 2).

**Table 6:** Recommendations for each adaptation method used by the nutritionists interviewed.

Item	*N*° of respondents^1^	Mean	Mode	Minimum	Maximum
Multiple step-up diets					
Average number of days to the finishing diet	19	19.26	21.00	14.00	30.00
Initial level of roughage (% of DM)	19	38.76	30.00	25.00	60.00
Number of adaptation diets used	20	2.63	3.00	2.00	4.00
Number of days per diet	16	7.97	7.00	5.00	12.00
Blending of two diets					
Average number of days to the finishing diet	7	13.29	6.00	6.00	21.00
Initial level of roughage (% of DM)	6	32.50	25.00	25.00	40.00
Finishing diet limited by quantity					
Average number of days to the finishing diet	2	21.00	21.00	21.00	21.00
Initial level of roughage (% of DM)	2	30.00	ND	25.00	35.00
Only one diet containing less energy than the finishing diet					
Average number of days to the finishing diet	5	18.50	21.00	15.00	21.00
Initial level of roughage (% of DM)	5	36.74	ND	25.00	57.50
DM – dry matter; ND – not determined.					

^1^ All respondents had either an answer for all possible choices or more than one answer per question.

### 3.6 General feeding and bunk management

#### 3.6.1 Mixers

The nutritionists interviewed (*n* = 34) indicated that 80.7% of their clients used truck-mounted mixers, 17.8% used a combination of stationary mixers and delivery trucks, 1.2% had only delivery trucks, and 0.3% of their clients did not use any type of mixer (Table 7). When it comes to the type of mixer, 80.1% of participants’ clients owned horizontal mixers, whereas 17.8% had vertical mixers. Nutritionists reported that 2.1% of their clients had no mixer at all (Table 7).

**Table 7:** Information on mixers provided by Brazilian nutritionists.

Item	*N*° of respondents^1^	Mean
Mixer (% of clients)	*n* = 34	
Truck-mounted mixer		80.75
Stationary mixer and delivery truck		17.78
Delivery truck		1.18
Does not use any mixer		0.29
Type of mixer used (% of clients)	*n* = 29	
Horizontal mixer		80.17
Vertical mixer		17.76
No mixer		2.07

^1^ All respondents had either an answer for all possible choices or more than one answer per question.

#### 3.6.2 Feed delivery and management

The participants (*n* = 34) indicated that 81.4% of their clients used a programmed feed delivery per pen; however, 18.6% of nutritionists’ clientele adopted continuous feed delivery (Table 8).

**Table 8:** Feeding management information provided by the Brazilian nutritionists.

Information	*N*° of respondents^1^	Mean	Mode	Minimum	Maximum
Feed delivery (% of clients)	*n* = 34				
Programmed delivery per pen		81.35			
Continuous delivery		18.65			
Feeding management					
Average mixing time for finishing diets (min)	34	6.10	5.00	3.00	15.00
Clients who add water to finishing diets (%)	24	41.31	100.00	5.00	100.00
Water added to finishing diets (%)	23	9.96	10.00	1.00	27.00
Daily feeding interval (h)	34	2.93	3.00	1.50	8.00
Bunk space per animal (cm)	34	34.08	30.00	15.00	50.00
Average water trough space per animal (cm)	31	5.10	2.50	2.00	10.00
Area per animal in a pen (m^2^)	34	14.09	15.00	7.00	25.00
Water trough cleaning (times per week)	34	2.84	3.00	1.00	7.00

Information	*N*° of responses^2^	% of responses			
Feeding	*n* = 34				
Cattle are fed one time daily	0	0.00			
Cattle are fed two times daily	1	2.94			
Cattle are fed three times daily	5	14.71			
Cattle are fed four times daily	26	76.47			
Cattle are fed five times daily or more	2	5.88			
Bunk management	*n* = 34				
Slick bunk	19	55.88			
1 to 3% orts	15	44.12			
Daytime feed bunk reading	*n* = 34				
Yes	34	100.00			
Nighttime feed bunk reading	*n* = 34				
Yes	18	52.94			
No	16	47.06			
Use of sprinklers in the pen	*n* = 32				
Yes	17	53.13			
No	15	46.88			

^1^ All respondents had either an answer for all possible choices or more than one answer per question. ^2^ Number of responses when nutritionists chose only one answer for a question.

The average mixing time for finishing diets in Brazil, obtained from 34 responses, was on average 6.1 min (minimum = 3.0; maximum = 15.0; and mode = 5.0), while the addition of water to finishing diets was adopted by 41.3% of participants’ clients, with 10.0% of water added on average (Table 8).

In addition, the average interval between feedings was 2.9 h (minimum = 1.5; maximum = 8.0; and mode = 3.0). When asked about specific feeding management, most participants (76.5%; n = 26) opted to feed cattle four times a day. In other cases, the preference is to feed cattle three times a day (14.7%; *n* = 5), followed by feeding cattle five times a day or more (5.9%; *n* = 2) and finally feeding cattle twice a day (2.9%; *n* = 1; Table 8).

For bunk management, the most common practice among Brazilian nutritionists was a slick bunk (55.9%; *n* = 19), followed by 1**–**3% orts (44.1%; *n* = 15). The unanimous choice of 34 participants (100.0%) was to read the bunks during the day. In addition, 52.9% of the participants (*n* = 18) also read bunks at night (Table 8).

The average bunk space per animal was 34.1 cm, and the pen area per animal, on average, was 14.1 m^2^ (Table 8). It was observed that 28 nutritionists have 18.8% of clients who adopted shading in pens as a strategy for animal welfare (data not shown). Among clients who adopted shading, 32.0% adopted natural shade, whereas 68.0%, preferred the use of artificial shade (data not shown). Seventeen (53.1%) out of 32 nutritionists reported the use of sprinklers in the pens (Table 8).

Thirty-two nutritionists (100%) indicated that their clients adopted vaccination against clostridium. Similarly, 84.4% of the participants (*n* = 27) reported that clients used vaccination against pneumonia. In addition, 31 nutritionists (100%) indicated that their clients dewormed cattle; however, the use of acaricide vaccination was cited only by 37.5% of the participants interviewed (*n* = 12). The average mortality rate, reported by 31 participants, was remarkably low at 0.51% (data not shown).

### 3.7 Cattle performance information

Information on cattle performance is shown in Table 9. The nutritionists surveyed reported that the predominant type of cattle in Brazilian feedlots are bulls, which are present in 89.7% of nutritionists’ feedlot clients, followed by heifers (25.6%), calves (23.2%), cull cows (13.9%), and steers (15.8%). Likewise, nutritionists declared that 83.5% of their clients feed Nellore cattle, while 54.3% feed crossbreds.

**Table 9:** Information on cattle performance provided by the nutritionists surveyed.

Information	Calf	Bull	Steer	Heifer	Cull cow	Nellore	Crossbred
Average initial age (mo)	8.79	22.58	26.00	19.98	57.58		
*n*=	14	30	7	25	19		
Average initial BW (kg)	220.67	384.81	380.00	304.18	380.26		
*n*=	15	33	9	26	19		
Average final BW (kg)	486.67	557.92	529.00	435.18	466.84		
*n*=	16	33	9	26	19		
Days on feed	117.33	106.18	91.50	92.46	67.63		
*n*=	15	33	9	26	19		
ADG (kg)	1.34	1.59	1.30	1.31	1.29		
*n*=	15	33	9	26	19		
Feed-to-gain ratio	6.28	7.00	7.39	7.51	8.77		
*n*=	12	29	9	22	15		
DMI (kg)	6.60	10.75	10.54	8.83	10.26	10.57	11.55
*n*=	13	32	8	25	18	25	24
DMI (% of BW)	2.38	2.32	2.30	2.40	2.46	2.27	2.41
*n*=	12	31	7	24	17	26	25
Clients who feed (%)	23.16	89.66	15.79	25.57	13.86	83.55	54.34
*n*=	16	32	9	26	20	31	31

BW – body weight; ADG – average daily gain; DMI – dry matter intake.

Regarding entry into the feedlot, 33 nutritionists indicated that 95.5% of their clients use some type of animal separation criteria. Most respondents (45.5%) opted for selection criteria based solely on animal weight, while 42.4% considered both weight and body condition. A smaller portion considered weight in conjunction with the breed (6.0%), and an even smaller percentage used criteria including weight, breed, and frame size (3.0%). Regarding cattle traceability, nutritionists reported that 55.4% of their clients adopted some traceability programs (data not shown). Furthermore, 87.0% of nutritionists’ clients marketed their animals as commodities, and only 21.9% participated in beef quality programs.

When asked average final weights of different types of cattle fed at their clients’ feedlots, nutritionists reported 557.9, 529.0, 466.8, 435.2, and 486.7 kg for bulls, steers, cull cows, heifers, and calves, respectively.

### 3.8 Recommended nutrient composition for finishing diets

#### 3.8.1 Fat

Table 10 shows that the surveyed nutritionists (*n* = 32) recommended a mean dietary fat of 5.2% (DM basis) in their finishing diets (mode = 4.5%). For the maximum dietary fat level recommended, the average was 6.8%, with a mode of 7.0%.

**Table 10:** Fat and protein recommendations for finishing diets used by Brazilian nutritionists.

Item	*N*° of respondents^1^	Mean	Mode	Minimum	Maximum
Recommended dietary fat (% of DM)	32	5.15	4.50	3.00	7.00
Maximum dietary fat recommended (% of DM)	32	6.81	7.00	5.00	8.00
Recommended level of CP (% of DM)	31	13.97	14.00	12.00	15.80
Recommended level of urea (% of DM)	31	1.07	1.20	0.40	1.50
Recommended true protein level for finishing diets (% DM)	29	10.04	10.00	4.50	12.60
Nutritionists who formulate for RDP	Yes *n* = 27	87.10			
	No *n* = 4	12.90			
RDP recommended for finishing diets (% of DM)	26	9.66	10.00	6.00	13.00
Main source of fat	*n* = 32				
Whole cottonseed	17	53.13			
Cottonseed cake	6	18.75			
DDG (Dry Distillers grains)	5	15.63			
Soybean grain	1	3.13			
Corn germ meal	1	3.13			
Rice bran	1	3.13			
Rumen-protected fat	1	3.13			
Primary source of protein	*n* = 31				
DDG (Dry Distillers grains)	12	38.71			
Soybean meal	7	22.58			
Cottonseed meal	4	12.90			
Peanut meal	3	9.68			
Whole cottonseed	2	6.45			
Cottonseed cake	2	6.45			
WDG (Wet Distillers grains)	1	3.23			
Secondary source of protein	*n* = 31				
DDG (Dry Distillers grains)	11	35.48			
Cottonseed cake	5	16.13			
Peanut meal	4	12.90			
Whole cottonseed	4	12.90			
Soybean meal	4	12.90			
Cottonseed meal	3	9.68			

DM, dry matter; CP, crude protein; RDP, rumen degradable protein. ^1^ All respondents had either an answer for all possible choices or more than one answer per question. ^2^ Number of responses when nutritionists chose only one answer for a question.

Regarding the main sources of fat (*n* = 32), whole cottonseed was cited by 53.1% of participants (*n* = 17), followed by cottonseed cake (18.8%; *n* = 6), DDG (15.6%; *n* = 5), soybean grain, corn germ meal, rice bran, and rumen-protected fat, each with 3.1% of the responses.

#### 3.8.2 Protein

For crude protein (CP), the average level recommended by the nutritionists was 14.0% (*n* = 31; Table 10), with an average urea level of 1.1% (mode = 1.2%; DM basis). Additionally, the recommended true protein level averaged 10.0% (*n* = 29). When asked about formulating using rumen-degradable protein (RDP; *n* = 30), 87.1% of the nutritionists stated they used it, whereas 12.9% did not. The recommended RDP level was 9.7% (*n* = 26; DM basis), with variations between 6.0 and 13.0%.

The DDG was cited by 38.7% of nutritionists as the primary source of protein in finishing diets, followed by soybean meal (22.6%), cottonseed meal (12.9%), peanut meal (9.7%), whole cottonseed (6.5%), cottonseed cake (6.5%), and WDG (3.2%).

#### 3.8.3 Major minerals

The average concentration of calcium recommended by the nutritionists for finishing diets was 0.60% of diet DM. Regarding phosphorus, potassium, and sodium concentrations, participants recommended 0.30, 0.64, and 0.27% of diet DM, respectively. For sulfur and magnesium, the average recommendations were 0.21 and 0.20% of diet DM, respectively (Table 11).

**Table 11:** Mineral and vitamin recommendations provided by nutritionists (DM basis).

Item	*N*° of respondents^1^	Mean	Mode	Minimum	Maximum
Major mineral (%)					
Calcium	25	0.60	0.90	0.25	0.90
Phosphorus	26	0.30	0.30	0.15	0.90
Potassium	27	0.64	0.80	0.10	0.99
Sodium	26	0.27	0.20	0.10	0.90
Sulfur	24	0.21	0.20	0.10	0.80
Magnesium	25	0.20	0.10	0.10	0.50
Trace mineral (mg/kg of diet)					
Iron	9	34.78	20.00	13.00	70.00
Zinc	25	57.69	60.00	30.00	90.00
Cobalt	24	0.52	0.40	0.10	1.20
Copper	25	14.05	15.00	4.00	25.00
Selenium	26	0.29	0.30	0.10	1.00
Iodine	25	0.69	0.50	0.50	1.20
Manganese	25	30.67	40.00	9.80	70.00
Vitamin (IU/kg)					
A	19	2626.58	2200.00	1200.00	5000.00
D	14	332.86	275.00	175.00	500.00
E	21	30.78	30.00	12.00	55.00

DM, dry matter; IU, international units. ^1^ All respondents had either an answer for all possible choices or more than one answer per question.

#### 3.8.4 Trace minerals

The concentrations of trace minerals in finishing diets indicated by the nutritionists were on average: 34.8, 57.7, 0.5, 14.1, 0.3, 0.7, and 30.7 mg/kg of diet DM for Iron, Zinc, Cobalt, Copper, Selenium, Iodine, and Manganese, respectively (Table 11).

#### 3.8.5 Vitamins

Nutritionists, when providing recommendations for vitamin supplementation in finishing diets, suggested average concentrations of 2,626.6 IU/kg for vitamin A, 332.9 IU/kg for vitamin D, and 30.8 IU/kg for vitamin E (Table 11).

#### 3.8.6 Feed additives

Most nutritionists (*n* = 30) reported that 100% of their clients use some type of feed additive in finishing diets (data not shown). The primary feed additive indicated by the interviewed nutritionists was monensin sodium, which was recommended by 70.0% of participants (*n* = 21), with a recommended level of inclusion of 26.0 mg/kg (DM basis), on average. Four nutritionists (13.3%) reported the use of monensin sodium + virginiamycin as additives of choice, and 2 participants (6.7%) indicated the use of salinomycin (6.7%; Table 12). For the second most used additive, virginiamycin was cited by 31.0% of the participants, with a mean inclusion level recommended of 21.8 mg/kg (DM basis). Additionally, adsorbents of mycotoxins were used by 49.6% (*n* = 26) of the participants (data not shown).

**Table 12:** Feed additive recommendations for finishing diets used by the Brazilian nutritionists.

Item	*N*° of responses^1^	% of responses	Recommended level	Mode	Minimum	Maximum
Primary feed additive used	*n* = 30					
Monensin sodium (mg/kg of DM)	21	70.00	26.05	25.00	20.00	30.00
Monensin sodium + Virginiamycin (mg/kg of DM)	4	13.33	23.13	25.00	15.00	25.00
Salinomycin (mg/kg of DM)	2	6.67	12.00	12.00	12.00	12.00
Monensin sodium and peptides blend	1	3.33	–	–	–	–
Monensin sodium + Functional oil	1	3.33	–	–	–	–
Lasalocid	1	3.33	–	–	–	–
Secondary feed additive used	*n* = 29					
Virginamycin	9	31.03	21.78	25.00	1,500	25.00
Monensin sodium	4	13.79	26.00	25.00	24.00	30.00
Monensin sodium + Virginiamycin	3	10.34	19.17	–	15.00	25.00
Tannins	3	10.34	4.17	-	1.50	7.00
Yeasts	3	10.34	–	–	–	–
Functional oil + enzymes	2	6.90	–	–	–	–
Lasalocid	1	3.45	–	–	–	–
Salinomycin	1	3.45	–	–	–	–
Zinc Bacitracin	1	3.45	–	–	–	–
Blend of essential oils	1	3.45	–	–	–	–
Peptides blend	1	3.45	–	–	–	–
DM–dry matter						

^1^ Number of responses when nutritionists chose only one answer for a question.

### 3.9 Problems reported by nutritionists

#### 3.9.1 Health problems

The most frequently highlighted health issues reported by 32 nutritionists included respiratory diseases in general, pointed out by 59.4% of the participants, followed by hoof-related problems, such as laminitis, which was mentioned by 15.6% of the respondents, clostridiosis and mycotoxins (12.5%), acidosis (6.3%), mycotoxin intoxication (3.1%), and an isolated case of fracture (3.1%; Table 13).

**Table 13:** Major health problems and challenges faced by Brazilian nutritionists to put into practice their nutritional recommendations.

Item	*N*° of responses^1^	% of responses
Major health problems	*n* = 32	
Respiratory diseases in general	19	59.38
Laminitis	5	15.63
Clostridiosis and mycotoxins	4	12.50
Acidosis	2	6.25
Mycotoxin intoxication	1	3.13
Bullying (fracture)	1	3.13
Major challenges	*n* = 32	
Employee training	14	43.75
Administration and management	14	43.75
Equipment precision and availability	3	9.38
Owner Engagement	1	3.13

^1^ Number of responses when nutritionists chose only one answer for a question.

#### 3.9.2 Major challenges

Fourteen nutritionists surveyed (43.8%) indicated that lack of trained employees was the most significant challenge for implementing their nutritional recommendations; the same number reported administration and management as the most challenging issue (43.8%; Table 13). The equipment precision and availability were mentioned by 9.4% of the participants, whereas owner engagement was cited in a smaller proportion by 3.1% of the nutritionists interviewed.

## 4 Discussion

### 4.1 General information

In the present survey, the results show that the 36 nutritionists interviewed play a crucial role, responsible for approximately 79.8% of the cattle from feedlots slaughtered in 2023. This percentage is higher than that reported in previous studies, which averaged 76.7% in Silvestre and Millen (4). Compared to the results of the first survey conducted by Millen et al. (1), 14 years ago, there is a significant decrease in the proportion of nutritionists serving feedlots with less than 5,000 head per year, from 71.0 to 45.7%. On the other hand, compared to the previous survey (4), there is a significant increase in the proportion of nutritionists now servicing feedlots with an average capacity of more than 20,000 animals, from 5.6 to 20.0%. Visits to feedlots are conducted, on average, every 35.4 days, approximately 9 days longer than the 26.3-day interval reported by Oliveira and Millen (2), indicating that the recommendations are being successfully implemented, increasing the interval between visits to realign practices in feedlots.

Of the nutritionists participating in the current survey, 22.2% worked as independent consultants, which is the only category that showed an increase compared to other options and it is higher than in previous surveys reported by Millen et al. (1), Oliveira and Millen (2), Pinto and Millen (3) (9.1%), and Silvestre and Millen (4) (5.6%). The position of feedlot manager increased from 3.0% in 2016 (3) to 11.1% in 2023. Regarding the time in practice, 91.7% of the participants have been practicing for more than 10 years, following the upward trend observed in recent studies reported by the previous Brazilian surveys. It’s noteworthy to mention that more than 50% of the nutritionists interviewed owned at least a master’s degree. Only two of the nutritionists surveyed possessed only a bachelor’s degree in animal science.

### 4.2 Information on grains and energy levels

The primary source of grains used in finishing diets for feedlot cattle has predominantly been corn (91.7%), maintaining its position as the top choice throughout the surveys conducted (1–4). In 2021, 97.2% of the interviewed nutritionists indicated corn as the main grain source, and in 2023, this percentage remained high at 91.7% (Table 1); however, it shows a movement toward greater inclusion of sorghum in finishing diets [8.3% vs. 2.8%; (4)]. Sorghum increased from 79.1% in 2021 to 85.7% in 2023 as the second most used option by nutritionists.

The corn used has consistently been led by a preference for flint types over the years, representing 97.1% of the responses in the present study. Regarding grain processing methods, it was noted that finely ground still holds its position as one of the most adopted options, representing 30.6% of the responses. However, this method now shared nutritionists’ preference with two others: coarsely ground and high-moisture storage (re-hydrated), both also corresponding to 30.6% of the responses. Conversely, there was a sharp decrease in the percentage of nutritionists who exclusively adopted the method of only cracked grains over the years, reducing from 57.6% (2) to merely 2.8% of the nutritionists in this survey (Table 1).

In the context of this survey, there was a notable preference for high-moisture corn grain silage; by summing high-moisture storage (re-hydrated) and high-moisture harvesting and storage (harvested wet), it represented the first choice of 36.1% of nutritionists’, and the second choice of 34.3% of the participants (Table 1). Based on the presented data, it is evident that nutritionists and feedlot owners are committed to improving their decisions regarding grain processing to enhance starch availability in diets. There is still a long way to spread out over the country methods such as re-hydration of corn grains; however, in the very first Brazilian survey, none of the nutritionists reported the use of high-moisture as the primary grain processing method adopted (1). Literature indicates that flint corn varieties benefit more from intensive grain processing methods because of their lower starch availability in comparison to dent types (26).

The inclusion of grains in finishing diets remained predominant in the 51–65% range, chosen by 44.4% of nutritionists. However, diets with 71–80% grain inclusion, which had grown in importance with 30.3% in 2016 (3) and 33.3% in 2019 (4), decreased to 13.9% in 2023. In contrast, 30.6% of nutritionists now opted for diets with 36–50% grain inclusion, a huge increase when compared to the previous survey (5.6%) conducted by Silvestre and Millen (4). Regarding the level of concentrate in finishing diets, was observed an increase from 83.2 to 84.2% (Table 4). The percentage of nutritionists that recommended more than 81% concentrate in the finishing diets increased from 63.9% (4) to 77.8% in the last 4 years. Over the past 10 years, there has been a significant decrease in the percentage of nutritionists reporting concentrate inclusion levels below 81%, dropping from 57.6% (2) to 22.2% in this survey.

The TDN remained the most indicated energy unit, representing 50.0% of the recommendations. However, there has been a progressive decrease in the use of TDN over the years by feedlot nutritionists: 83.8% (1), 78.8% (2), 69.7% (3), and 52.8% (4). On the other hand, the use of NEg has become more prominent in the responses, registering an increase in its use in the last decade [33.3% compared to 6.5% in (1)]. Since the analysis of feedstuffs in private labs has become more accessible in Brazil, nutritionists have been using nutritional models, such as NASEM and LRNS, in their mechanistic approach, which simulates rumen fermentation and calculates NEg more precisely. Moreover, TDN is expressed as a percentage rather than in Mcal, which is the typical unit for expressing energy content. In terms of TDN, the energy content of finishing increased [76.8% vs. 74.7%; (4)] in the last 4 years. Despite the reduction in the inclusion of grains, the increase in the energy content of finishing diets at Brazilian feedlots is explained by the greater use of high-moisture corn and by the increase of 1% in the concentrate level, represented predominantly by the higher inclusion of coproducts.

Regarding the nutritional models and main sources of information on feed energy values, the BCNRM or NASEM (6) was mentioned by 31.3% of the nutritionists interviewed, closely followed by the RLM, mentioned by 28.1% of the participants. The NRC (24) received 9.4% of the responses, which indicates that 40.3% of the Brazilian nutritionists interviewed use some of the NRC models.

### 4.3 Use of coproducts

It was reported by 34 feedlot nutritionists that 92.7% of their incorporate some sort of coproducts into their finishing diets, reflecting a continuous increase over the years: 79.7% (1), 82.4% (2), 70.6% (3), 82.3% (4).

The most recommended coproduct by these professionals (*n* = 34) was citrus pulp pellets, mentioned by 26.5% of the participants, with an average inclusion of 26.1%, a lower level when compared to previous surveys: 33.8% (1), 40.0% (2), 29.2% (3), 27.8% (4). Whole cottonseed which has been the primary concentrate coproduct over the last 14 years, was cited as the preference of 20.6% of the nutritionists. The DDG (20.6%) and cottonseed cake (17.6%) were the coproducts that replaced whole cottonseed in the last 4 years. Levels of inclusion of those coproducts are shown in Table 3.

In this survey, it is noteworthy that the use of DDG significantly increased. Among the secondary concentrate coproducts, 34.3% of the surveyed nutritionists also preferred it. It is important to note that DDG appeared for the first time in the previous survey conducted by Silvestre and Millen (4) (8.3% of the responses and an average inclusion of 15.0% DM basis) due to the expansion of the grain milling industry for ethanol in Brazil. The use of higher inclusions of DDG, and other coproducts of the ethanol industry such as WDG, explains the reduction in the inclusion of grains in the finishing diets without negatively impacting the energy content of finishing diets.

### 4.4 Roughage sources and levels and fiber analysis methods

The average inclusion level of roughage in finishing diets, as recommended by the nutritionists, reached 15.7% (DM basis). This index indicates a decrease compared to the previous survey conducted by Silvestre and Millen (4) (16.8%), resulting in a trend of reducing roughage incorporation into finishing diets since the first Brazilian survey [26.4%; (1)].

With the expansion of large feedlot operations in Brazil, there was a gradual transition from fresh feeds, such as fresh-chopped sugarcane and sugarcane bagasse, to conserved feeds, such as corn silage, which was cited by 55.9% of nutritionists as the primary roughage source. Moreover, the use of corn silage has contributed to an increase in the energy content of finishing diets in Brazil, as it has a higher energy value than grass silage, sorghum silage, and sugarcane silage mentioned in this research (7). However, due to the increase in corn silage production costs in the last 4 years in Brazil, the use of grass silage increased, and it is already the second most used roughage source in the Brazilian feedlots (Table 4), indicated by 20.6% of nutritionists.

The survey by Pinto and Millen (3) reported a notable change in preferences regarding fiber analysis methods, revealing for the first time the preference of Brazilian nutritionists by peNDF (67.7% of the nutritionists). From 2016 onwards, the use of peNDF was further increased: to 80.6% (4) and 85.2% in this survey. With the increase in the energy content of finishing diets over the past decade, the use of peNDF has become more precise than NDF for monitoring particle size in feedlot diets. The average inclusion level of peNDF in the diet, which reached 13.6%, is in line with the recommendations of Goulart and Nussio (8), who suggested that finishing diets for Nellore cattle in Brazil should contain between 10 and 18% dietary peNDF.

### 4.5 Cattle adaption methods and receiving programs

The use of methods to receive cattle has gained increasing popularity among feedlot nutritionists due to its importance in allowing cattle to recover from physiological and immune factors and physical stressors associated with handling and transport procedures (9). These events often result in negative impacts on cattle health and productivity during the feedlot period (10).

About a decade ago, a study found that 42.4% of the nutritionists interviewed did not adopt any type of receiving program (2). However, in this present survey, only 15.2% of the participants did not use any type of receiving program. The evolution of receiving programs relies on the increased energy content of finishing diets in Brazil and the fact that cattle usually arriving from dry-season pastures come to the feedlot hungry and immune depressed. The receiving program conducted in the feedlot pen, where the bunks contained a mix of roughage and concentrate feedstuffs, remained the most adopted among feedlot nutritionists (48.5%; Table 5) when compared to the previous survey [44.4%; (4)].

As evidenced by previous research (1–4), the most widely adopted adaptation method by nutritionists was still the multiple step-up diets (Tables 5, 6), chosen by 20 nutritionists (58.8% of the responses). In this method, an average of 2.6 diets was used, with an average of 8 days per diet; values close to those reported by Silvestre and Millen (4): 2.9 and 7.14, respectively. Additionally, the period required to adapt the animals using multiple step-up diets was 19.3 days, which was like the 19.2 days reported by Silvestre and Millen (4) and longer than the adaptation length reported in the previous surveys, including the very first one in 2009, where nutritionists reported, on average, adaptation length of 17.1 days (1). The constant increase in the energy content of finishing diets in Brazilian feedlots over the past 14 years may explain why nutritionists are adopting longer adaptation periods. However, despite the adaptation using the multiple step-up diets has become longer, it remains in line with the recommendation that feedlot cattle, both in Brazil and the USA, should not be adapted in less than 14 days to high-energy diets (11, 12). Finally, the surveyed nutritionists recommended an initial roughage concentration of 38.8% when adopting the multiple step-up diets, a value lower than previous recommendations, which were 54.7% (1), 50.5% (2), and 45.1% (3), but slightly higher than indicated by Silvestre and Millen (4), which was 36.9%. The use of corn silage as the primary source of roughage, combined with improved management practices over the last decade, has enabled nutritionists to increase the energy content of finishing diets. This was achieved by increasing the inclusion level of concentrate feedstuffs while reducing the inclusion of roughage sources. Based on these observations, cattle might require additional time to achieve the expected DMI necessary for the finishing diet.

The blending of two diets was the second method of adaptation of choice among the nutritionists interviewed (*n* = 7; 20.6%). However, Brazilian nutritionists recommending this method are adapting cattle, on average, for 13.3 days, which is below the minimum recommended adaptation length of 14 days mentioned by Brown et al. (11), Parra et al. (12) and Pereira et al. (13).

### 4.6 Mixers

Regarding clients who adopted truck-mounted mixers, as shown in Table 7, there has been a significant increase over the years, indicated by the fact that 80.7% of the nutritionist’s clients use this type of mixer. In 2009 this number was 40.5% (1), increased to 52.3% in 2013 (2), went up to 71.5% in 2016 (3) and to 79.0% in 2019 (4). On the other hand, the use of only delivery trucks decreased over the last 14 years, from 41.9 to 1.2% of nutritionist’s clients.

The increasing adoption of truck-mounted mixers also contributed to an increase in the percentage of nutritionists’ clients (81.4%, Table 8) who adopt programmed delivery per pen, which is 11.9% higher than reported in the last survey (4) and 35.8% higher than the 45.6% reported 14 years ago (1). As a result, the broader use of truck-mounted mixers and programmed delivery per pen allowed Brazilian nutritionists to increase the energy content of finishing diets. As discussed earlier, ruminal acidification is under better control when the amount of feed offered per pen is precisely controlled day-by-day and well mixed to avoid particle sorting.

### 4.7 Feeding management

The percentage of water added to the finishing diet (10.0%) has not varied much over the years compared to previous surveys [9.3%, (1); 11.0%, (2); 10.2%, (3); 12.0%, (4)]. However, the percentage of nutritionist’s clients who adopt this practice has increased over the years: 41.3% in this survey vs. 12.1% (1) and 32.1% (3). Since diets have become more energetic over the years, it usually leads to drier diets which often need water addition to be more palatable to the animals.

The average mixing time for finishing diets remains constant at 6.1 min (*n* = 34), coinciding with the value reported in the study by Silvestre and Millen (4). The current research showed that 44 nutritionists (76.5%) opted for four feedings daily, with an average interval between feedings of 2.9 h (*n* = 34). These values continue to be adopted with minor variations in the interval between hours [3.0 h, (1); 2.9 h (2, 3); 3.0, (4)]. Literature highlights improvements in feedlot performance when feeding Nellore cattle three to four times a day, compared to once or twice a day (14).

In this broad context, in bunk management (*n* = 34), it is observed that 55.9% of the nutritionists recommended the slick bunk practice, while 44.1% opted for a range of 1 to 3% orts. It is noteworthy to mention that compared to previous studies, the predominant practices were reversed, with 48.4% of the nutritionists adopting 1 to 3% of orts and 25.8% preferring the slick-bunk approach (1, 2). Furthermore, none of the nutritionists interviewed reported the adoption of orts greater than 3%, which is an evolution of the Brazilian feedlots that shows a reduction in feed waste.

Nutritionists indicated that animals have an average pen area of 14.1 m^2^, lower than reported in previous years [16.5 m^2^, (2); 15 m^2^, (3); 15.2 m^2^, (4)]. Regarding bunk space per animal, the numbers also pointed out to a reduction of 6.8 cm/animal compared to the study published by Oliveira and Millen (2). The use of sprinklers in feedlot pens was mentioned by 53.1% (*n* = 17) of nutritionists, representing an 8.7% increase compared to the previous survey [44.4%; (4)]. The reduced dust in a pen due to the use of sprinklers may contribute to lowering respiratory diseases, the primary health issue in feedlots in Brazil and North America (15), which may lead to improved cattle performance. Concerning thermal comfort, for the first time, nutritionists were asked about the use of shade for cattle, resulting in 28 responses reporting that only 18.8% of clients use some shade in the pens, of which 68% is artificial, and 32% is natural (*n* = 25; data not show). The Embrapa Sudeste has conducted research showing that the provision of artificial shade reduces water footprint and improves the efficiency of nutrient use (16).

Regarding health management (data not shown), all 32 respondents reported the use of vaccination against Clostridium. Similarly, vaccination against pneumonia showed considerable acceptance, with 84.4% (*n* = 27) adherence. When comparing these results with a previous study (4), a positive variation in both practices was observed: 97.2% adopted vaccination against Clostridium and 66.7% against pneumonia. Deworming of cattle was universally applied and adopted by all the 31 nutritionists who responded to this question. However, acaricide vaccination showed variation, being adopted by 37.5% (*n* = 12) of participants, consistent with previous findings by Silvestre and Millen (4). Regarding the average mortality rate, the results revealed a rate of 0.5%, slightly lower than the 0.6% reported by Silvestre and Millen (4).

### 4.8 Cattle performance information

Most nutritionists (95.5%; *n* = 33) indicated that their clients employ some criterion for selecting animals upon entry into the feedlot, with a noticeable balance between those who sort based solely on weight (45.5%) and those who opt for a combination of weight and body condition score (42.4%; data not shown). This change in selection criteria is evidenced by the fact that, in previous surveys, the option of only body weight was more prevalent among nutritionists [(1–4); 73.9%]. This demonstrates an evolution of Brazilian feedlots since nutritionists are more concerned with associating body weight and body condition score for pen formation to be more accurate to match nutritional requirements. On the other hand, nutritionists reported that only 55.4% of their clients’ cattle were part of some traceability program (data not shown), which is in agreement with previous surveys [57.2% (3); 57.9% (4)].

At Brazilian feedlots, 89.7% of nutritionists’ clients feed bulls, which agrees with previous studies (1–4). Moreover, bulls are more efficient than steers and heifers in terms of performance (17, 18), and currently are the best fit for the Brazilian beef production system that is focused on commodity animals. Additionally, the use of anabolic implants is prohibited in Brazil, providing another reason for choosing bulls over steers in feedlot operations. Nutritionists reported that 87% of their clients feed commodity cattle, and only 21.9% feed cattle for beef quality programs (data not shown).

Bulls are on average 22.6-month-old and weigh 384.8 kg at feedlot arrival, reaching an average final weight of 557.9 kg after 106.2 days in the feedlot (Table 9). However, compared to the first study by Millen et al. (1), animals are currently being slaughtered heavier (500.7 vs. 557.9 kg). The mature weight of Nellore cattle, the predominant breed in Brazil and at Brazilian feedlots (83.6% of nutritionists’ clients; Table 9), ranges between 560 and 580 kg (27). Nutritionists have been taking advantage of the feed efficiency of cattle slaughtered at lighter weights when compared to Nellore’s mature weight (28). Likewise, the increase in diet energy content makes carcass fat deposition more consistent, increasing the potential of Nellore bulls to deposit a minimum amount of fat to match the Brazilian market requirements (5 mm on carcasses’ back fat), and decreasing the use of steers and heifers.

### 4.9 Recommended nutrient composition for finishing diets

#### 4.9.1 Fat and protein

The recommended dietary fat of 5.2% in this survey did not change when compared to Silvestre and Millen (4) (5.2%). However, the maximum dietary fat recommended increased 3% [6.8% vs. 6.6%; (4)]. Anyhow, the maximum dietary fat recommended by Brazilian nutritionists has been increasing over the last 14 years. Millen et al. (1) reported that nutritionists recommended 6.1% as the maximum dietary fat content. Nevertheless, the levels of fat inclusion in finishing diets, regardless of average or maximum content, are within the guidelines suggested by Oldick and Firkins (19), who warned that dietary fat levels above 7.0% (DM basis) might adversely affect fiber digestion in the rumen. Over the past 14 years, protein recommendations have not changed much and remained relatively stable when compared to previous studies (1–4): 14.0% for crude protein level (DM basis), 1.1% (DM basis) for urea, 10.0% (DM basis) for true protein level, and 9.7% (DM basis) for RDP.

Regarding the main fat sources (*n* = 32), whole cottonseed was still the first choice of 53.1% of participants; however, its use has been decreasing over the last 4 years since nutritionists started recommending other fat sources such as DDG and cottonseed cake more often. The DDG was also the first choice of nutritionists as a protein source for the first time in the series of Brazilian surveys, demonstrating its versatility and wide application in ration formulations.

#### 4.9.2 Minerals

Nutritionists participating in the survey indicated in Table 11 that the average calcium and phosphorus concentrations in finishing diets were 0.60 and 0.30% (DM basis), which is consistent with the recommendations of Silvestre and Millen (4) (0.63 and 0.30%, respectively).

In contrast, the potassium concentration has decreased since the first study by Millen et al. in 2009 (0.64% vs. 0.82% DM basis). One of the reasons for that is the lower level of forage used in the finishing diets nowadays compared to 2009. The trace minerals recommended by the nutritionists did not vary much compared to the last 2 surveys (3, 4), and they still meet the requirements according to NASEM (6).

#### 4.9.3 Vitamins

The vitamin concentrations in this survey were higher than those reported by Millen et al. (1). For vitamin A, Brazilian nutritionists suggested a recommendation of 2,626.6 IU/kg (min = 1,200 mode = 2,200 max = 5,000), slightly higher than the value reported by Silvestre and Millen (4), which was 2,583.5 IU/kg. However, this level is still lower than that reported by American nutritionists, who recommended 4,715 IU/kg (15). The inclusion of more roughage in finishing diets in Brazil compared to the United States may explain these discrepancies in vitamin A recommendations, as tropical roughage contains a greater amount of this vitamin in its composition (20). For vitamin D, it is important to note that this survey presents a value of 332.96 IU/kg, a significant increase compared to the previous survey, which recorded 291.6 IU/kg (4). In the same way, vitamin E also showed an increase, from 24.2 IU/kg (4) to 30.8 IU/kg in this survey.

#### 4.9.4 Feed additives

Monensin, highlighted as the most adopted feed additive by feedlot nutritionists (Table 12; *n* = 30), is an ionophore mentioned in 70% of the responses in this survey, as well as was first option of nutritionists in previous surveys in Brazil (1–4) and USA (15). A growing trend in recent years reveals that the combination of monensin and virginiamycin in finishing diets (13.3%; *n* = 30) has become popular, driven by promising results related to increased carcass weight (21). However, based on the survey results, we cannot say with certainty that all nutritionist clients who use virginiamycin as a secondary feed additive also employ monensin sodium as a primary feed additive. Conversely, Virginiamycin is cited as the second most used feed additive (31% of responses; *n* = 29).

### 4.10 Problems reported by nutritionists

#### 4.10.1 Health problems

Respiratory diseases in general, which were reported by 59.4% of participants in this survey, remained the main health problem in the Brazilian feedlots. However, the percentage of nutritionists indicating this problem was lower than that recorded in the last survey [71.4%; (4)]. This reduction may be related to the higher proportion of clients now using sprinklers in feedlot pens [53.1% vs. 44.4% in (4)] and the adoption of the pneumonia vaccination protocol [84.4% vs. 66.7% in (4)].

#### 4.10.2 Major challenges

Lack of trained employees and administration and management were mentioned as the major challenges for nutritionists to put into practice their nutritional recommendations. It is noteworthy that the administration and management were reported in a lower proportion when compared to the previous survey [61.1%; (4)]. This implies that although Brazilian feedlots have increased their investments in feeding technologies, there is still a need to invest in the training of employees to ensure the skills to handle these technologies. In addition, the challenge of administration and management involves the synchronization of all feedlot processes, which includes coordination of nutritional recommendations with feeding delivery, mixing time, feeding frequency, and bunk management, among others.

## 5 Conclusion

A comprehensive understanding of nutrition and management practices in Brazilian feedlots over the last 14 years was reported in this study, highlighting significant improvements in feed management.

The Brazilian feedlots are still evolving, evidenced by the greatest use of truck-mounted mixers and programmed delivered per pen by nutritionists’ clients ever. As a result, nutritionists have shifted bunk management to the minimum possible waste. These practices certainly allowed feedlot nutritionists to increase the energy content of finishing diets, which resulted in the greatest use of peNDF as well as monitoring the amount of fiber available for rumination in the diets. Such advances certainly have played a significant role in increasing the use of Nellore bulls and their final weight in Brazilian feedlots.

The increasing use of coproducts was one of the main highlights of this survey, demonstrating the concern of nutritionists to use economically viable and sustainable sources capable of providing quality nutrients. The DDG has become one the most widely used concentrate coproducts, especially as a protein source. Furthermore, the results of this survey on grain processing methods indicated that nutritionists are looking to optimize starch utilization as the high-moisture corn has become, for the first time in Brazilian history, the preference of most nutritionists interviewed. Despite the diversification in grain processing practices, the rehydration of grains (corn and sorghum) appears to be the method that Brazilian nutritionists will still use in the next couple of years to continuously improve starch digestion in feedlot cattle.

Thus, the data collected point to a notable change in the diets and management practices of Brazilian feedlots. This evolution reflects an adaptation to the needs of the sector, as well as a growing commitment to efficiency and sustainability. These trends point to a promising future for feedlots in Brazil and highlight the continued need for research and innovation to drive feedlot operations to advanced practices.

## Data Availability

The raw data supporting the conclusions of this article will be made available by the authors, without undue reservation.

## Footnotes

^1^ https://pt.surveymonkey.com/
