# Supplementary material for: A snapshot of nutritional recommendations and management practices adopted by feedlot cattle nutritionists in Brazil in 2023
Source: Front Vet Sci. 2025 Jun 4;12:1518571. doi: 10.3389/fvets.2025.1518571 (PMC12175091; doi:10.3389/fvets.2025.1518571)
Supplement: Supplementary file 1 [file Data_Sheet_1.PDF]

A snapshot of nutritional recommendations and management practices adopted by feedlot cattle nutritionists in Brazil in 2023

General information n=11

General information on grains and concentrate recommended in finishing diets n=13

Use of coproducts in finishing diets n=5

Sources and levels of forage n=5

Adaptation methodes n=7

Mixers n=6

Feeding management n=7

Animal management n=15

Information about diet formulation n=17

Information about sources for nutritional recommendations n=2

Additional questions n=12

Total = 100 questions

GENERAL INFORMATION

1. What is your ID number?

2. What is the nature of your consulting practice?

a) Independent

b) Nutritional consulting company

c) Corporate feed manufacturing company

d) University

e) Other (please specify)

3. Years of practice?

a) 2 years or less

b) 2 to 5 years

c) 5 to 8 years

d) 8 to10 years

e) More than 10 years

4. What is your terminal degree?

a) Bachelor of Science
b) Specialization courses in cattle nutrition
c) Master of Science
d) Ph.D.
e) Other (please specify)

5. State (s) where the degree was obtained (check more than one If necessary):

a) Goiás
b) Mato Grosso
c) Mato Grosso do Sul
d) Minas Gerais
e) Paraná
f) Rio Grande do Sul
g) São Paulo
h) Rio de Janeiro
i) Other (please specify)

6. Approximate number of cattle per year that your practice serves:

7. Average number of cattle in feedlots that your practice serves:

a) Less than 1,000
b) 1,001 to 5,000
c) 5,001 to 10,000
d) 10,001 to 20.000
e) More than 20.000

8. State (s) in which yours clients are located (check more than one if necessary):

a) Bahia
b) Goiás
c) Mato Grosso
d) Minas Gerais
e) Pará
f) Paraná

g) Rio Grande do Sul
h) São Paulo
i) Tocantins
j) Other (please specify)

9. Do you have clients in other countries? Please mark the area (s).

a) None
b) Paraguay
c) Argentina
d) Uruguay
e) U.S.A.
f) Other (please specify)

10. Percentage of your practice in foreign countries (if applicable):

11. On average, how often do you visit your clients (days):

GENERAL INFORMATION ON GRAINS AND CONCENTRATE RECOMMENDED IN FINISHING DIETS

1. Primary grain used by your clients in finishing diets (please mark one):

a) Corn
b) Sorghum
c) Barley
d) Wheat
e) Other (please specify)

2. If you marked CORN on the previous question, mark the grain type most used:

a) Flint
b) Dent

3. Second grain used by your clients in finishing diets (please mark one):

a) Corn
b) Sorghum

c) Barley

d) Wheat

e) Other (please specify)

4. Primary grain processing method used by your clients in finishing diets (please mark one):

a) Steam-flaking

b) Dry-rolling

c) High-moisture harvesting and storage (re-hydrated)

d) High-moisture harvesting and storage (harvested wet)

e) Only cracked

f) Finely ground

g) Coarsely ground

h) No processing (whole shelled grain)

i) Other (please specify)

5. If you answered “only cracked”, “finely ground” or “coarsely ground” in the previous question, what is the grain size of the corn media used by your clients (mm)?

6. Second grain processing method used by your clients in finishing diets (please mark one):

a) Steam-flaking

b) Dry-rolling

c) High-moisture harvesting and storage (re-hydrated)

d) High-moisture harvesting and storage (harvested wet)

e) Only cracked

f) Finely ground

g) Coarsely ground

h) No processing (whole shelled grain)

i) Other (please specify)

7. Level of inclusion of GRAIN in the finishing diets (% DM):

a) 20 to 35%

b) 36 to 50%

c) 51 to 65%

d) 66 to 80%

e) More than 81%

8. Level of inclusion of CONCENTRATE ingredients in the finishing diets (% DM):

a) Less than 55%
b) 56 to 70%
c) 71 to 80%
d) 81 to 90%
e) More than 91%

9. What is the energy unit used to formulate your diets?

a) T.D.N. (Total Digestible Nutrients)
b) NEg (Net Energy for Gain)
c) ME (Metabolizable Energy)
d) NFC (Non-fibrous Carbohydrates)
e) Other (please specify)

10. According to the previous question, what is the energy concentration recommended for finishing diets?

11. Average DM content of your finishing diets:

12. How often are feedstuffs that compose your finishing diets sent to chemical analysis?

13. Which one do you recommend to your customers for the determination of the dry matter of the diet and the ingredients?

a) Microwave
b) Koster
c) Oven
d) Air Fryer
e) None
f) Other (please specify)

COPRODUCT USE IN FINISHING DIETS

1. **Percentage of your clients who use coproducts in finishing diets (ZERO if none):**

2. **Primary coproduct used by your clients in finishing diets (please mark one):**

a) Citrus Pulp, pellets
b) Whole Cottonseed
c) Corn gluten feed (Refinazil®)
d) Soybean Hulls
e) WDG (Wet Distiller's grains)
f) DDG (Dry Distiller's grains)
g) Cottonseed hulls
h) Other (please specify)

3. **According to the previous question, what is the level of inclusion of this primary coproduct in finishing diets (% DM):**

4. **Second coproduct used by your clients in finishing diets (please mark one):**

a) Citrus Pulp, pellets
b) Whole Cottonseed
c) Corn gluten feed (Refinazil®)
d) Soybean Hulls
e) WDG (Wet Distiller's grains)
f) DDG (Dry Distiller's grains)
g) Cottonseed hulls
h) Other (please specify)

5. **According to the previous question, what is the level of inclusion of this primary coproduct in finishing diets (% DM):**

ROUGHAGE SOURCES AND LEVELS

1. **Primary roughage source used by your clients in finishing diets (please mark one):**

a) Sugarcane Bagasse
b) Sugarcane Bagasse, Hydrolyzed
c) Corn Silage
d) Grass Silage

e) Sorghum Silage
f) Hay
g) Fresh Chopped Sugarcane
h) Cottonseed prod
i) Peanut shells
j) Other (please specify)

2. Second roughage source used by your clients in finishing diets (please mark one):

a) Sugarcane Bagasse
b) Sugarcane Bagasse, Hydrolyzed
c) Corn Silage
d) Grass Silage
e) Sorghum Silage
f) Hay
g) Fresh Chopped Sugarcane
h) Cottonseed prod
i) Peanut shells
j) Other (please specify)

3. Typical range of inclusion of roughage in finishing diets (% DM):

4. Method of fiber analysis considered in formulation:

a) Crude Fiber
b) Neutral Detergent Fiber (NDF)
c) Acid Detergent Fiber (ADF)
d) Physically effective NDF
e) None

5. According to the previous question, what is the fiber concentration recommended in finishing diets (% DM)?

ADAPTATION METHODS

1. Which method do you use for adapting cattle to finishing diets (Check one)?

a) None
b) Two-ration blending
c) Multiple step-up diets
d) Final diet limited by quantity
e) Only one diet containing less energy than the final diet
f) Other (please specify)

2. If you use TWO-RATION BLENDING:

a) What is the average number of days to the final diet?
b) What is the initial level of roughage (% DM)?

3. If you use MULTIPLE STEP-UP DIETS:

a) What is the average number of days to the final diet?
c) What is the initial level of roughage (% DM)?
d) Number of step-up diets used:
e) Number of days per diet:

4. If you use FINAL DIET LIMITED BY QUANTITY:

a) What is the average number of days to the final diet?
b) What is the level of roughage (% DM)?

5. If you use ONLY ONE DIET CONTAINING LESS ENERGY THAN THE FINAL DIET:

a) What is the average number of days to the final diet?
b) What is the initial level of roughage (% DM)?

6. Which receiving program(s) is the most used by your clients before adopting any adaptation method?

a) None
b) Bunk containing only roughage
c) Bunk containing hay plus concentrate
d) Some days on pasture
e) Pasture plus concentrate
f) Pasture + bunk containing roughage and concentrate
g) Other (please specify)

7. **According to the previous question, what is the average number of days in the receiving program?**

MIXERS

1. **What is the percentage of your clients who use (ZERO if none)?**

a) Only delivery truck?
b) Stationary mixer/delivery truck?
c) Truck-mounted mixers?
d) Do not use any type of mixer?

2. **What is the percentage of your clients who use (ZERO if none)?**

a) Programmed delivery per pen?
b) Continuous delivery (feed offer per pen not controlled)?

3. **Mixer design used by most of your clients (% of clients, ZERO if none):**

a) Horizontal mixer?
b) Vertical mixer?
c) None?

4. **Average mixing time used by your clients for the finishing diets (minutes):**

5. **Percentage of your clients who add water in the mixed diet (ZERO if none):**

6. **Average level of water inclusion (for responses different of ZERO on the previous question):**

FEEDING MANAGEMENT

1. **Number of times cattle are fed per day:**

a) 1
b) 2
c) 3
d) 4

e) 5or more

2. Daily feeding interval in hours (ZERO if none):

3. Do your clients' feedlots conduct daytime feed bunk reading to manage feed leftovers?

a) Yes

b) Non

4. Do your clients' feedlots conduct nighttime feed bunk reading?

a) Yes

b) Non

5. What is the bunk management adopted by most of your clients?

a) None

b) Clean-bunk

c) 1 to 3% orts

d) 3 to 5% orts

e) 5 to 10% orts

f) Other (please specify)

6. What is the bunk space for at your client's operations (linear meters/animal)?

7. What is the pen space at your client's feedlot (density: m^2/animal)?

CATTLE MANAGEMENT AND INFORMATION

1. Percentage of your clients that include their cattle in some traceability program (ZERO if none):

2. What percentage of your clients sort cattle into outcome groups (BW, BCS, etc.; ZERO if none):

3. Most common method of sorting (% of clients; ZERO if none):

a) Weight only:

b) BCS:

c) Weight and BCS:

d) Ultrasound:

e) Other (specify):

4. Average initial age when calves, bullocks, steers, heifers and cull cows start on feed in the feedlots of your clients (NA, if not applicable to your practices):

a) Calves:

b) Bullocks

c) Steers:

d) Heifers:

e) Cull Cows:

5. Average initial BW when calves, bullocks, steers, heifers and cull cows start on feed in the feedlots of your clients (NA, if not applicable to your practices):

a) Calves:

b) Bullocks

c) Steers:

d) Heifers:

e) Cull Cows:

6. Average final BW for calves, bullocks, steers, heifers and cull cows in the feedlots of your clients (NA, if not applicable):

a) Calves:

b) Bullocks:

c) Steers:

d) Heifers:

e) Cull Cows:

7. Average days on feed for calves, bullocks, steers, heifers and cull cows in the feedlots of your clients (NA, if not applicable):

a) Calves:

b) Bullocks:

c) Steers:

d) Heifers:

e) Cull Cows:

8. What is the typical average daily gain for calves, bullocks, steers, heifers and cull cows in the feedlots of your clients (NA, if not applicable)?

a) Calves:

b) Bullocks:

c) Steers:

d) Heifers:

e) Cull Cows:

9. On average, what is the DMI in KILOGRAM obtained in the feedlots of your clients for calves, bullocks, steers, heifers and cull cows (NA, if not applicable)?

a) Calves:

b) Bullocks:

c) Steers:

d) Heifers:

e) Cull Cows:

10. On average, what is the DMI in % of BW obtained in the feedlots of your clients for calves, bullocks, steers, heifers and cull cows (NA, if not applicable)?

a) Calves:

b) Bullocks:

c) Steers:

d) Heifers:

e) Cull Cows:

11. What is the typical feed to gain ratio for calves, bullocks, steers, heifers and cull cows obtained in the feedlots of your clients (NA, if not applicable)?

a) Calves:

b) Bullocks:

c) Steers:

d) Heifers:

e) Cull Cows:

12. **On average, what is the DMI in KILOGRAM obtained in the feedlots of your clients for Nellore and Crossbreds (NA, if not applicable)?**

a) Nellore:

b) Crossbreds:

13. **On average, what is the DMI in % of BW obtained in the feedlots of your clients for Nellore and Crossbreds (NA, if not applicable)?**

a) Nellore:

b) Crossbreds:

14. **Percentage of your clients that feed calves, bullocks, steers, heifers and cull cows (NA if not applicable):**

a) Calves:

b) Bullocks:

c) Steers:

d) Heifers:

e) Cull Cows:

15. **Percentage of your clients that feed Nellore and Crossbreds (NA if not applicable):**

a) Nellore:

b) Crossbred:

FORMULATION INFORMATION FOR FINISHING DIETS

1. **Recommended level of total fat in finishing diets (% of DM; NA if not applicable):**

2. **What is the maximum level of total fat in finishing diets (% of DM; NA if not applicable)?**

3. **The main source of fat used in finishing diets by your clients?**

a) Rumen-protected fat

b) Whole Cottonseed

c) Cottonseed hulls

d) DDG

e) Soybean grain

f) Other (please specify):

4. Protein Levels:

a) Protein level recommended for finishing diets (% of DM):

b) Urea level recommended for finishing diets (% of DM):

c) True protein level recommended for finishing diets (% DM)

5. Primary protein source used by your clients in finishing diets (please mark one):

a) Soybean meal

b) Cottonseed meal

c) Peanut meal

d) Whole cottonseed

e) DDG (Dry Distillers Grains

f) WDG (Wet Distillers Grain)

g) Other (please specify)

6. Second protein source used by your clients in finishing diets (please mark one):

a) Soybean meal

b) Cottonseed meal

c) Peanut meal

d) Whole cottonseed

e) DDG (Dry Distillers Grains

f) WDG (Wet Distillers Grain)

g) Other (please specify)

7. Do you formulate for degraded intake protein (DIP)?

a) Yes

b) No

8. If yes, what level of DIP do you recommend (% of DM)?

9. What are the recommendations of major minerals in finishing diets that you practice (in% of DM; NA if not applicable to your practices):

a) Calcium:

b) Phosphorus:
c) Potassium:
d) Sodium:
e) Chlorine:
f) Sulfur:
g) Magnesium:

10. What is the recommendation of trace minerals in finishing diets that you practice (in ppm; NA if not applicable to your practices):

a) Iron:
b) Zinc:
c) Cobalt:
d) Molybdenium:
e) Copper:
f) Selenium:
g) Iodine:
h) Manganese:

11. Vitamin supplementation in finish diets (ZERO, if none; NA if not applicable to your practices):

a) Recommended vitamin A level (IU / kg):
b) Recommended vitamin D level (IU / kg):
c) Recommended vitamin E level (IU / kg):

12. Feed Additives (NA if not applicable):

% of clients who use some type of feed additive:

13. Primary feed additive used

a) Monensin
b) Lasalocid
c) Virginiamycin
d) Monensin + Virginamycin
e) Narasin
f) Salinomycin

g) Functional oils
h) Functional oils + enzymes
i) Yeast
j) Tannins
k) Flavomycin
l) Other (please specify)

14. According to the previous answer, what is the recommended inclusion level (ppm or mg / kg DM)?

15. What is the second type of food additive most used by clients?

a) Monensin
b) Lasalocid
c) Virginiamycin
d) Monensin + Virginamycin
e) Narasin
f) Salinomycin
g) Functional oils
h) Functional oils + enzymes
i) Yeast
j) Tannins
k) Flavomycin
l) Other (please specify)

16. According to the previous answer, what is the recommended inclusion level (ppm or mg / kg DM)?

17. Percentage of your clients who use mycotoxin adsorbent in their diets?

INFORMATION RESOURCES FOR NUTRITIONAL RECOMMENDATIONS

1. What are the main source(s) of information on nutrient requirements of beef cattle that you use in formulating nutritional recommendations?

a) None
b) NRC (1976)

c) NRC (1984)
d) NRC (1996)
e) BCNRM (2016)
f) CNCPS Cornell
g) BR – Corte
h) RLM
i) Personal Information
j) Private company source
k) Other (please specify)

2. What are the main source(s) of scientific or recent information that you use?

a) Brazilian Journal of Animal Science
b) Journal of Animal Science
c) DBO (a Brazilian beef magazine)
d) Globo Rural
e) Feed & Food
f) Other (please specify)

MAJOR HEALTH PROBLEMS

1. Are there other nutrients, in addition to those listed above, particularly minerals and vitamins, which you supplement in termination diets? If so, which one?

a) Non
b) Yes (please, specify)

2. What are the major health problems faced by your clients?

a) Acidosis
b) Laminitis
c) Bloat
d) Respiratory diseases in general
e) Clostridiosis
f) Other (please specify)

3. Prophylactic management adopted by the majority of yours clients when the animals arrive in the feedlot:

a) Clostridium vaccination:

Yes

No

b) Pneumonia vaccination:

Yes

No

c) Vermifuge:

Yes

No

d) Acaricide:

Yes

No

4. What is the mortality rate?

5. Regarding water in the feedlot: How often the water trough is cleaned in the week? (NA if not applicable):

a) 1x

b) 2x

c) 3x

d) Daily

e) NA

6. What is the water through space per animal (cm/animal)?

7. Using sprinklers in the pen:

a) Yes

b) No

8. Percentage of your clients who implement shading in pens for animal comfort:

9. When there is shading in the pens, is it:

a) Natural

b) Artificial

10. What are the main challenges faced by you to put into practice your nutritional recommendations in the feedlots of your clients?

a) Logistics
b) Employees training
c) Availability and precision of equipment
d) Management
e) Other (please specify)

11. How are your customers' animals marketed at the time of slaughter?

a) Commodity:
b) Quality programs:

12. We would like you to suggest any questions for the next editions of the survey:
